# Anti-nociceptive properties of cardiopulmonary baroreceptors in patients with chronic back pain

**DOI:** 10.3389/fpain.2025.1593939

**Published:** 2025-07-25

**Authors:** Yuto Iwakuma, Jennifer Liu, Davina A. Clonch, Megan E. Gangwish, Christopher M. Lam, Seth W. Holwerda

**Affiliations:** ^1^ Department of Anesthesiology, University of Kansas Medical Center, Kansas City, KS, United States; ^2^ College of Osteopathic Medicine, Kansas City University, Kansas City, MO, United States; ^3^ Department of Cell Biology and Physiology, University of Kansas Medical Center, Kansas City, KS, United States; ^4^ KU Diabetes Institute, University of Kansas Medical Center, Kansas City, KS, United States

**Keywords:** baroreflex, chronic pain, cardiopulmonary, descending inhibition, sympathetic nerve activity (SNA)

## Abstract

**Introduction:**

Reduced pain perception following a persistent noxious stimulus during a study session (short-term habituation) is believed to be partially mediated by descending inhibitory mechanisms, although these mechanisms have not been fully elucidated. We examined the hypothesis that cardiopulmonary baroreceptor would significantly increase short-term habituation in chronic back pain (CBP) patients.

**Methods:**

A short-term habituation protocol was utilized that involved 1-sec pulses (×10) at 105% heat pain threshold on the anterior forearm at 0.5 Hz. Cardiopulmonary baroreceptor unloading was performed via lower body negative pressure (LBNP) that reduces central venous pressure to elicit a reflex increase in sympathetic nerve activity.

**Results:**

Short-term habituation was observed in young, healthy participants (*n* = 11), as indicated by a reduction in subjective pain ratings across the 10 repetitive heat pulses (−42% ± 29, *P* < 0.01, *n* = 11). Short-term habituation was also observed in CBP patients (−32% ± 30, *P* < 0.01, *n* = 12). Cardiopulmonary baroreceptor unloading via LBNP significantly reduced pain ratings across the 10 repetitive heat pulses in CBP patients compared with supine control (patient positioned in LBNP chamber but without a reduction in pressure) and upright sitting (chair), as indicated by a more negative area under the curve index (LBNP: −16.3 ± 4.1; Control: −14.4 ± 2.6; Upright sitting: −15.1 ± 4.1, *P* = 0.02). However, LBNP-mediated reductions in pain ratings were selective to CBP patients with more severe symptoms, i.e., neuropathic pain (LBNP: −14.7 ± 2.1; Control: −12.8 ± 1.4; Upright sitting: −12.1 ± 1.2, *P* = 0.04), whereas no effect of LBNP was observed in young, healthy participants (*P* = 0.83). In support, CBP patients with neuropathic pain exhibited significantly elevated mechanical pressure pain threshold during LBNP (*P* = 0.04).

**Conclusions:**

Together, these findings demonstrate an association between cardiopulmonary baroreceptor unloading and a reduction in pain perception during repetitive noxious stimuli in CBP patients, particularly among CBP patients with greater pain severity.

## Introduction

Descending pain inhibitory pathways attenuate the perception of pain following persistent noxious stimuli that cannot be avoided, such as post-operative pain. The general class of adaptive processes whereby perceived pain is attenuated following a persistent noxious stimulus is termed habituation. Reduced or absent habituation within a testing session (short-term habituation) has been reported in chronic pain conditions such as chronic back pain (1, 2), fibromyalgia (3–5), and migraine (6). Therefore, efficient habituation may describe a descending pain inhibitory pathway important for protection against the development of chronic pain. However, experimental protocols demonstrating short-term habituation in patients with chronic pain that include methodology that may potentially translate to the clinical setting have been sparse. There is also inadequate knowledge regarding endogenous anti-nociceptive mechanisms that contribute to impaired short-term habituation in chronic pain.

Habituation to persistent or repetitive painful stimuli is mediated, at least in part, by descending pain inhibition (7). Descending pain inhibition includes input from arterial baroreceptors (8–10), which are both myelinated (A*δ*) and unmyelinated (C) afferent fibers with nerve terminals in the adventitia of the arterial wall that project to the nucleus tractus solitarius (NTS) in the brainstem (11, 12). Baroreceptors increase firing rate in response to mechanical stretch of the arterial wall during increases in arterial blood pressure (BP) and are critical for moment-to-moment regulation of BP via reflex changes in sympathetic and parasympathetic nerve activity (13, 14). Numerous studies in humans demonstrate reduced pain perception following activation of arterial baroreceptors (15, 16).

Nociception is also modulated by cardiopulmonary baroreceptors, which are located within the atria, ventricles, and pulmonary vasculature, and project to the nucleus tractus solitarius (NTS) in the brainstem via vagal afferents. However, in contrast to arterial baroreceptors, available data suggest pro- rather than anti-nociceptive properties of cardiopulmonary baroreceptor activation (17–21). For example, direct electrical stimulation of the vagal nerve carrying cardiopulmonary baroreceptor afferents reduces thermal pain threshold (i.e., increased pain sensitivity) (21). In line with these findings are higher subjective ratings of a painful stimulus (venipuncture) following fluid ingestion, which increases central blood volume and activates cardiopulmonary baroreceptors (19). Moreover, increased pain perception in response to cardiopulmonary baroreceptor activation via posture maneuvers, e.g., sitting to supine, passive leg elevation, have been reported (18, 19), although changes in pain perception in response to posture maneuvers are not observed in all studies (20, 22). Taken together, findings suggest increased nociception and pain in response to activation (loading) of cardiopulmonary baroreceptors.

There are limited studies that have directly examined pain modulation via unloading of cardiopulmonary baroreceptors, such as when central venous pressure is reduced from baseline. Importantly, no studies have examined pain modulation via unloading of cardiopulmonary baroreceptors in patients with chronic pain. This becomes important when considering clinical management of BP in chronic pain because hypertension is a common comorbidity (23–25) and central venous pressure can be reduced with diuretic anti-hypertensive medications (26, 27). Cardiopulmonary baroreceptor unloading is commonly performed in a laboratory setting via lower body negative pressure (LBNP), which induces a shift in blood volume from the upper to lower body, thereby reducing central venous pressure. The distinct advantage of LBNP is isolation of baroreflex mechanisms from confounding autonomic reflexes related to a change in posture, such as vestibular modulation of sympathetic nerve activity (28–30), and muscle contraction that occurs during orthostatic weight loading.

In the present study, we examined short-term habituation using a protocol involving thermal stimulation and LBNP with several different methods of calculating short-term habituation that was first tested for repeatability in young, healthy adults. Next, short-term habituation was tested in patients with chronic back pain (CBP) and further examined during modulation of cardiopulmonary baroreceptors via LBNP with the hypothesis that cardiopulmonary baroreceptor unloading would elicit analgesia and thereby enhance short-term habituation.

## Materials

All experimental procedures and protocols conformed to the Declaration of Helsinki and were approved by the Institutional Review Board at the University of Kansas Medical Center (STUDY0014659). Each participant received a verbal and written explanation of the study objectives, measurement techniques, and risks and benefits associated with the investigation prior to providing written informed consent on the initial visit.

### Participants

The short-term habituation reliability study included 11 participants (6 men and 5 women) that were young and healthy (24 ± 1 years) without history of chronic pain. The LBNP study included 19 participants; 7 participants were young and healthy without history of chronic pain (3 men, 4 women; 23 ± 1 years), and 12 participants (3 men and 9 women) had CBP and were primarily middle-aged (54 ± 11 years). Sample size was based on resources available for conducting the study. Patients with CBP were recruited from clinics at the University of Kansas Medical Center (KUMC) using a KUMC research participant registry that includes patients that volunteered to be contacted for research studies. Recruitment was performed primarily via email correspondence. Medical students that were young and healthy without history of chronic pain or cardiovascular disease were recruited internally via social media at the University of Kansas Medical Center to participate in reliability studies of short-term habituation and another group of young, healthy participants were recruited to participate in studies examining short-term habituation during LBNP. Importantly, the purpose for the young, healthy group of participants in LBNP studies was to initially determine the efficacy of short-term habituation during LBNP and not intended for direct comparisons with CBP patients because of the significant age difference. Inclusion criteria included men or women, age 18–79 years, chronic back pain (>3 months), or no history of chronic pain (young, healthy participants). Exclusion criteria included current diagnosis of cancer, active infection, or unstable BP in past 3 months (e.g., change in antihypertensive medications). CBP patients with history of hypertension were not excluded; however, participants were instructed to refrain from anti-hypertensive medications on the day of study visit to minimize any potential influence on cardiovascular parameters. CBP patients were instructed to maintain their normal regimen for all other medications, which are described in Table 1.

**Table 1:** Chronic back pain (CBP) patient demographics.

Variable	All CBP (*n* = 12)	Nociceptive (*n* = 6)	Neuropathic (*n* = 6)	*P*-value
Clinical questionnaires				
painDETECT (PD-Q)	15 ± 9	9 ± 6	21 ± 9	0.01
BPI-sf	58 ± 18	49 ± 21	66 ± 11	0.11
CSQ-CAT	8 ± 6	6 ± 2	9 ± 8	0.40
ODI	30 ± 8	29 ± 10	32 ± 4	0.50
PANAS	49 ± 13	47 ± 16	52 ± 10	0.53
PROMIS anxiety	48 ± 8	48 ± 7	49 ± 10	0.75
PROMIS depression	48 ± 7	47 ± 8	49 ± 6	0.61
Chronic pain				
Duration, years	14 ± 15	16 ± 19	12 ± 12	0.59
Sensory testing				
Heat pain threshold, °C	43.0 ± 1.3	42.9 ± 1.4	43.1 ± 1.5	0.83
Pressure pain threshold, kgf	240 ± 94	208 ± 90	271 ± 95	0.27
Pressure pain threshold + 4°C water, kgf	270 ± 106	264 ± 116	275 ± 106	0.87
Conditioned pain modulation, *Δ* kgf	116 ± 25	131 ± 26	101 ± 12	0.03
Pressure pain tolerance, kgf	431 ± 142	498 ± 159	364 ± 91	0.10
Ambulatory 24-hr BP				
Systolic BP, mmHg	117 ± 7	115 ± 7	119 ± 14	0.58
Diastolic BP, mmHg	72 ± 9	71 ± 6	73 ± 13	0.69
Resting cardiovascular variables				
Systolic BP, mmHg	129 ± 13	122 ± 3	135 ± 17	0.08
Diastolic BP, mmHg	84 ± 11	81 ± 4	87 ± 15	0.35
Spontaneous cardiac BRS, ms/mmHg	6.3 ± 3.6	5.2 ± 2.5	7.3 ± 4.4	0.35
Medications				
Anti-hypertensives, *n*	6	5	1	0.08
Prescription opioids, *n*	3	1	2	1.00
Non-steroidal anti-inflammatory, *n*	4	1	3	0.54
Muscle relaxants, *n*	5	3	2	1.00
Anticonvulsants, *n*	4	2	2	1.00
Psychotropic medications, *n*	5	3	2	1.00

Values are means ± SD. *P*-values are nociceptive vs. neuropathic. BPI-sf, brief pain inventory short form; CSQ-CAT, coping strategies questionnaire-catastrophizing subscale; ODI, Oswestry disability index; PANAS, the positive and negative affect schedule; PROMIS, patient-reported outcomes measurement information system; BP, blood pressure; BRS, spontaneous cardiac baroreflex sensitivity. Comparisons were made using independent *t*-tests.

### Experimental measurements

#### Clinical pain assessments

Pain severity was determined using the painDETECT (PD-Q) screening tool, which is suitable for CBP and has high sensitivity (85%) and specificity (80%) (31). The PD-Q is designed to discriminate between nociceptive and neuropathic components of chronic pain (maximum score: 38, minimum score: −1; scores ≤12 indicating low probability of neuropathic pain) and includes 7 sensory symptom items (burning, tingling, pain at light touch, sudden pain attacks, pain at cold or heat, numbness, and pain at slight pressure), a temporal item (persistent with or without attacks), and one spatial item (radiating pain to other areas of the body). Patients with a neuropathic pain component(s) (e.g., radiating pain, allodynia) generally suffer more severely than those without (31). Pain severity was also assessed with the Brief Pain Inventory (BPI-sf), which is one of the most common tools for assessing clinical pain and is valid for patients with CBP (32). The BPI-sf is a numeric rating scale (0–10) consisting of 12 items that assess severity of pain (current symptoms, symptoms on average, and range of pain intensity) and its impact on daily life (how pain interferes with their general activity, mood, mobility, work, relationships, sleep, and enjoyment of life). Pain catastrophizing was assessed with the coping strategies questionnaire-catastrophizing subscale (CSQ-CAT), and functional disability was assessed with the Oswestry Disability Index (ODI) (also named Low Back Pain Disability Questionnaire) where 0%–20% indicates minimal disability, 21%–40% indicates moderate disability, 41%–60% indicates severe disability, and 81%–100% indicates bed-bound or patient is exaggerating symptoms. Because questionnaire scores can have considerable variability (painDETECT, BPI-sf, PANAS, CSQ-CAT, ODI), the score for each CBP patient was an average value between 2 separate measures taken approximately 1 week apart.

#### Cardiovascular variables

Heart rate (HR) was determined from lead II of a three-lead ECG and beat-to-beat BP was monitored via finger photoplethysmography using the Finapres® NOVA (Finapres Medical Systems, Enschede, the Netherlands). Stroke volume was estimated via the Modelflow method (33), and total peripheral resistance (TPR) was calculated as mean arterial pressure (MAP) divided by the product of stroke volume and heart rate. Absolute values of BP were taken from auscultatory BP at the brachial artery during periods of resting baseline.

#### Thermal pain stimulus

Thermal stimuli were administered using a Medoc Thermal Sensory Analyzer (TSA-II, Medoc, Inc., Ramat, Isreal), which uses dedicated graphic based software to control the pattern, duration, rate of increase/decrease, and intensity of a thermal stimulus from a 30 × 30 mm Peltier thermistor probe to the nondominant ventral forearm. To reach threshold, the heat stimulus increased at a consistent rate and participants were instructed to press a hand-held button the moment when the intensity of the heat stimulus changed from a comfortable heat to an uncomfortable heat, or when the intensity would be rated as 1 on a scale of 0–10. Therefore, the stimulus intensity that was achieved was above the perception threshold and below the pain tolerance.

#### Short-term habituation

**Figure 1:**
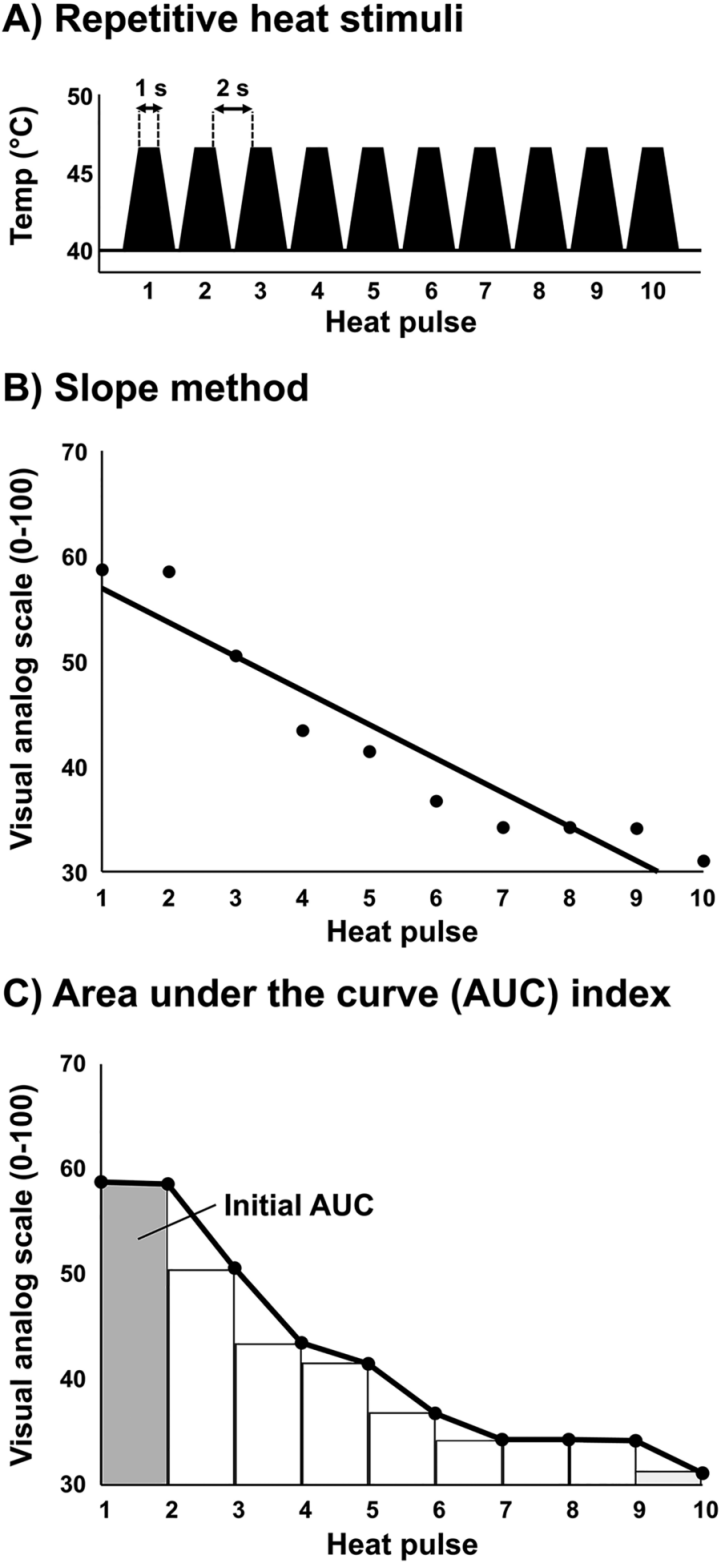
Short-term habituation protocol. **(A)** Ten repetitive heat pulses were administered at an intensity of 105% of each participant's previously determined heat pain threshold. **(B)** The ten repetitive heat pulses were divided into 9 subintervals using a right end point approximation for quantification of area under the curve (AUC). The initial AUC is represented by the first interval (average pain ratings of first and second heat pulses) and is divided by the total AUC to determine short-term habituation (AUC index). Example data provided in panels **(B,C)** are average data for patients with CBP (*n* = 12).

Short-term habituation was achieved using a repetitive heat stimulation protocol like that used for inducing wind-up (pain facilitation) (34–36). However, instead of using a relatively high heat stimulus intensity that can elicit wind-up (35), a lower stimulus intensity equal to 105% of heat pain threshold was utilized (−44°C–46°C) because preliminary studies indicated that this stimulus intensity would consistently produce a subjective pain rating of ∼50 on a scale of 0–100 among young, healthy participants. Each sequence of thermal stimuli was programmed to include 10 successive pulses of a 2-s duration and 1-s interval between pulses at a heat intensity equivalent to 105% of the average pain threshold delivered from a 40° C baseline (Figure 1A). The baseline of 40° C overcomes the problem of fluctuations in skin temperature during the study visit and differences in skin temperature between study visits and between participants. The rate of increase and decrease in temperature (temp/sec) was 105% threshold minus baseline (40°C) divided by two (on average ∼2.0°C–2.5°C/sec). Consequently, participants with higher heat pain threshold received a higher rate of change in temperature so that the thermode stimulator would reach the target temperature while also maintaining a separation of 2 s between the peak of each heat pulse. Participants were asked to rate their perceived pain intensity at the peak of each thermal stimulus on a visual analog scale of 0–100 (Figure 1B). Importantly, participants were instructed that each successive pulse would be somewhat less, more, or the same as their threshold intensity in a randomized fashion, and therefore were blinded to the intensity of each pulse. However, each pulse was programmed to reach the same temperature (105% of the average pain threshold) across the 10 successive pulses. The probe was moved to a different non-overlapping site on the forearm for each subsequent sequence of pulses.

#### Mechanical pressure pain threshold

Pressure pain threshold was assessed using a hand-held mechanical pressure algometer (Algomed) on the left upper trapezius, approximately 2 cm from the acromioclavicular joint. The hand-held algometer is both a reliable and valid process to quantify subjective pain as objective pain (37). The pressure stimulus increased at a consistent rate for all participants based on the rate graph visualized on the monitor, and participants were instructed to press a hand-held button the moment when the intensity of the pressure stimulus changed from a comfortable pressure to an uncomfortable pressure, or when the intensity would be rated as 1 on a scale of 0–10. Three pressure pain threshold measures were performed at the same anatomical location under each condition (upright sitting, supine, and LBNP −10 mmHg), and the 3 measures under each condition were averaged to represent pressure pain threshold.

#### Conditioned pain modulation (CPM)

The conditioned pain modulation (CPM) paradigm was used to test diffuse noxious inhibitory control (DNIC), which is one of the main descending pain inhibition pathways (38). A standard CPM paradigm was performed as previously described (39): Pressure pain threshold was assessed using a hand-held mechanical pressure algometer (Algomed) on the left upper trapezius, approximately 2 cm from the acromioclavicular joint. Three pressure pain threshold measures were averaged at baseline 5 min before the conditioning stimulus and 15 s into the conditioning stimulus. The conditioning stimulus was a cold pressor test (4°C water) using the right hand. Participants were instructed to press a hand-held button the moment when the intensity of the pressure stimulus changed from a comfortable pressure to an uncomfortable pressure, or when the intensity would be rated as 1 on a scale of 0–10.

#### Ambulatory 24-hr BP monitoring

Noninvasive 24-hr ambulatory BP, which is regarded as the gold standard for the prediction of risk related to BP (40, 41), was obtained using oscillometric SpaceLabs 90207 monitors (SpaceLabs Healthcare, Snoqualmie, WA) (42). Monitors were programmed to obtain BP readings at intervals of 30 min during the day from 0600 to 2200 hours and at night every 60 min from 2200–0600 hours. At least 10 daytime readings and 5 nighttime readings and at least 80% successful readings of planned measurements over the 24 hours were required (43).

#### Experimental protocol

On the first visit to the laboratory, participants received verbal explanation of the study and provided written informed consent. Participants were familiarized with thermosensory testing and performed the conditioned pain modulation (CPM) paradigm. Next, participants were instrumented for heart rate and finger photoplethysmography (beat-to-beat BP) and experienced the LBNP protocol. Finally, CBP patients were instrumented with a 24-hr ambulatory BP monitor to take home and completed clinical pain assessments (self-report questionnaires) via REDCap on a home computer. Two CBP patients declined the 24-hr ambulatory BP monitor. Within two weeks of the initial visit, participants returned for thermosensory testing. Heat pain threshold was determined followed by three trials of short-term habituation. Participants were then instrumented for heart rate and finger photoplethysmography (beat-to-beat BP) and performed the LBNP protocol described below. All experiments were performed in a dimly lit room at an ambient temperature of 22–24°C.

#### Lower body negative pressure protocol

The lower portion of the participant's body below the iliac crest was enclosed in a box-like chamber to allow the application of negative pressure to the lower body in the supine position as previously described (44). Participants underwent a 5 min quiet resting period, then the vacuum motor controlling the chamber pressure was turned on. To minimize the potential for cardiovascular responses related to anticipation of LBNP, a countdown was not provided to the participant of when LBNP would begin after the quiet baseline period. The LBNP chamber pressure was reduced at a rate of −0.5 mmHg/s until reaching −10 mmHg and was continuously monitored. The rationale for performing only low intensity LBNP at −10 mmHg was to reduce central venous pressure (∼3 mmHg) and elicit significant reflex increases in sympathetic nerve activity (∼25%) without eliciting major changes in steady-state BP and heart rate (45–47). Each participant underwent 3–4 trials of LBNP, and each trial was either −10 mmHg or 0 mmHg (control condition without vacuum seal) in a randomized fashion separated by at least 5 min. Regardless of the condition, the vacuum motor was on during each trial to provide a consistent auditory stimulus. LBNP exposure was limited to 2 min per trial to isolate autonomic from humoral responses (48), and the number of trials were limited to 3–4 because preliminary studies (familiarization visits) indicated that longer duration in the supine position for LBNP often became uncomfortable for CBP patients.

### Data analysis

#### Short-term habituation

Several approaches were used to analyze short-term habituation. First, significant main effects and interactions were tested among the ten repetitive heat pulses and the conditions (upright sitting, supine, supine with LBNP). Second, the last-minus-first method was used where the average of the first two subjective pain scores (scale 0–100) are subtracted from the average of the last two subjective pain scores (49). Third, the slope method was used where a linear regression was fitted to the series of subjective pain scores (*X*-axis: trial number, *Y*-axis: pain score) and the slope of the linear regression represents the magnitude of habituation (34). Finally, to further quantify short-term habituation, the ten repetitive heat pulses were divided into 9 subintervals using a right end point approximation for quantification of area under the curve (AUC). The initial AUC is represented by the first interval (average pain ratings of first and second heat pulses) and is divided by the total AUC to determine short-term habituation. The AUC index accounts for the baseline heat pain sensitivity because total AUC is relative to the initial starting point, as described below:

InitialAUC=((P1+P2)/2)×(2−1)

Where *P* = heat pulse pain score

TotalAUC=(((P1+P2)/2)×(2−1))+(((P2+P3)/2)×(3−2))+....

(((P9+P10)/2)×(10−9))

AUCindex(short−termhabituation)=(initialAUC/totalAUC)×100

#### Cardiovascular responses to LBNP

Hemodynamics were examined during LBNP at the onset when chamber pressure began to decrease and then reached −10 mmHg (30 s duration), steady state LBNP at −10 mmHg when sensory testing was performed (60 s duration), and during the recovery period after chamber pressure returned to 0 mmHg (120 s duration). Values for total peripheral resistance, cardiac output, and MAP were calculated as a percent of baseline, which was the 2-min resting period before the onset of LBNP.

#### Cardiac baroreflex sensitivity

Spontaneous cardiac baroreflex sensitivity (BRS) was estimated during the 10-min resting baseline period (supine) using the sequence technique (Nevrokard software, Izola, Slovenia). Briefly, sequences of three or more consecutive beats where systolic BP and R-R interval change in the same direction were identified as baroreflex sequences (50). A linear regression was applied to each individual sequence, and an overall average was calculated for a measure of spontaneous cardiac baroreflex sensitivity. Only sequences where *R*^2^ was >0.85 were accepted. Gains were determined for all sequences combined, i.e., ups and downs.

#### Statistical analysis

Differences in subjective pain scores over the course of 10 repetitive stimuli and between conditions were tested using two-way repeated measures analysis of variance (ANOVA), and when data were not normally distributed, differences were tested using Generalized Estimating Equations (GEE). Differences in calculated values for short-term habituation between conditions were tested using a one-way ANOVA and were tested using Friedman repeated measures ANOVA on ranks when data were not normally distributed (Shapiro–Wilk). Intraclass correlation (ICC), which is a widely used index of test-retest reliability analysis (51), was used to determine the reliability of short-term habituation measurements between separate visits among young, healthy participants. ICC estimates and their 95% confidence intervals were based on a single-rating, absolute agreement, 2-way random-effects model. ICC values less than 0.5 = poor reliability, between 0.5 and 0.75 = moderate reliability, between 0.75 and 0.9 = good reliability, and greater than 0.9 = excellent reliability (51, 52). Chi-squared tests were used to examine group differences in categorical variables. When normality failed, Kruskal–Wallis one-way ANOVA (ranks) tests were used. Statistical analysis was performed using IBM ® SPSS software. Data are reported as mean ± standard deviation unless otherwise noted. Statistical significance was set at *P* < 0.05.

## Results

### Participant characteristics

Six CBP patients (57 ± 12 years of age) were considered to have nociceptive pain components and 6 CBP patients (51 ± 11 years of age) were considered to have neuropathic pain components, based on painDETECT (PD-Q) threshold of 12 (<12 likely nociceptive). CBP patients had 14 ± 15 years of chronic pain (Table 1). The duration of chronic pain was not significantly different between nociceptive pain patients vs. neuropathic pain patients (*P* = 0.59). High variability in duration of chronic pain was a result of two outliers: One nociceptive pain patient had chronic pain for 55 years and one neuropathic pain patient had chronic pain for 36 years. The duration range of chronic pain for the remaining 10 CBP patients was 4–12 years.

Neuropathic pain patients demonstrated reduced conditioned pain modulation compared with nociceptive pain patients (*P* = 0.03). Indeed, 3 patients with neuropathic pain symptoms did not demonstrate conditioned pain modulation. However, no significant difference in baseline pressure pain threshold was observed between nociceptive and neuropathic pain groups (*P* = 0.27) (Table 1). Group differences were determined using independent t-tests. CBP patients were on average not hypertensive based on ambulatory 24-hr BP (Table 1), although it should be noted that 2 CBP patients declined the ambulatory 24-hr BP cuff. One CBP patient with features of neuropathic pain declined the ambulatory 24-hr BP cuff had elevated supine resting BP (147/92 mmHg) and thus potentially hypertensive. Among all other CBP patients, resting systolic BP tended to be higher in the supine position compared with upright sitting, although not statistically significantly (129 ± 13 vs. 123 ± 14 mmHg, *P* = 0.12). Resting diastolic BP in the supine position was not significantly different from upright sitting (84 ± 11 vs. 83 ± 13 mmHg, *P* = 0.61) in CBP patients. In young, healthy participants, no significant differences were observed between resting supine and upright sitting for systolic BP (111 ± 4 vs. 112 ± 4 mmHg, *P* = 0.76) and diastolic BP (84 ± 11 vs. 83 ± 13 mmHg, *P* = 0.61).

### Reliability of short-term habituation in young, healthy participants (*n* = 11)

**Figure 2:**
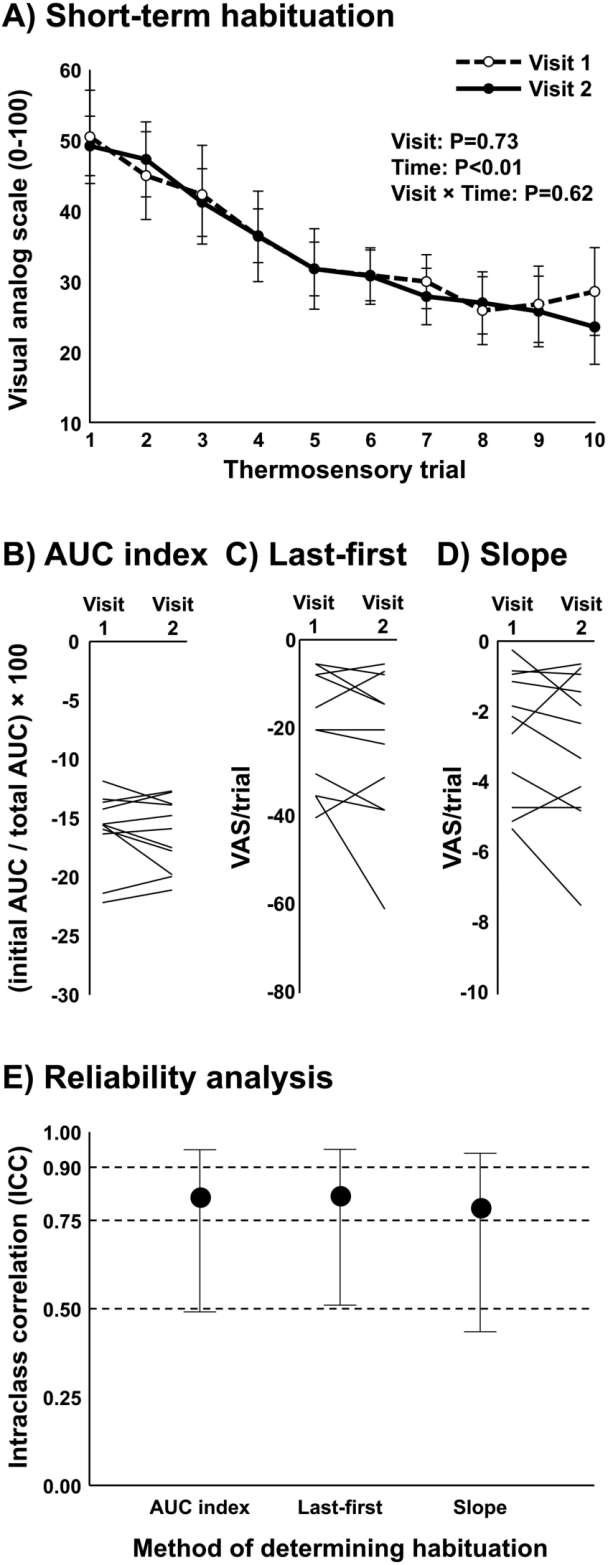
Reliability analysis of short-term habituation in young, healthy participants (*n* = 11). **(A)** Subjective pain ratings following ten repeated thermal stimuli at 105% threshold in the upright sitting position on separate visits within 1 week. Each participant was blinded to the intensity and was instructed that each trial would be somewhat less, more, or the same as their threshold intensity in a randomized fashion. Differences between time points and conditions were tested using a two-way repeated measures ANOVA. **(B–D)** Short-term habituation in each participant at visit 1 and visit 2 as determined by the area under the curve method (AUC index), last-first method, and the slope method. **(E)** Intraclass correlation ± 95% confidence intervals to test reliability of short-term habituation between visit 1 and visit 2 while using the AUC index method, last-first method, and the slope method.

Average heat pain threshold was 43.2 ± 1.3°C. The average subjective pain rating for 105% of threshold was 51 ± 22 (VAS 0–100). A significant reduction in subjective pain ratings over the ten repetitive heat pulses was observed (*P* < 0.01), indicating short-term habituation. Importantly, there was no significant difference between visits in the subjective pain ratings over the 10 repetitive heat pulses (*P* = 0.73) (Figure 2A). Differences between time points and conditions were tested using a two-way repeated measures ANOVA. Also, good reliability was observed between visits when short-term habituation was determined by the AUC index, last-minus-first method, and the slope method (Figure 2B). Intraclass correlation (ICC) ± 95% confidence intervals were used to test reliability. All ICC models were statistically significant (all *P* < 0.01). The subjective pain rating at the onset of the 10 repetitive heat pulses (initial AUC) at the first and second visits demonstrated excellent reliability (ICC = 0.94, 95% CI: 0.81–0.98) (data not shown in graph), in addition to the total AUC showing a strong correlation (R = 0.93, *P* < 0.001) and excellent reliability (ICC = 0.92, 95% CI = 0.71–0.98) (data not shown in graph).

### LBNP and short-term habituation in young, healthy participants (*n* = 7)

**Figure 3:**
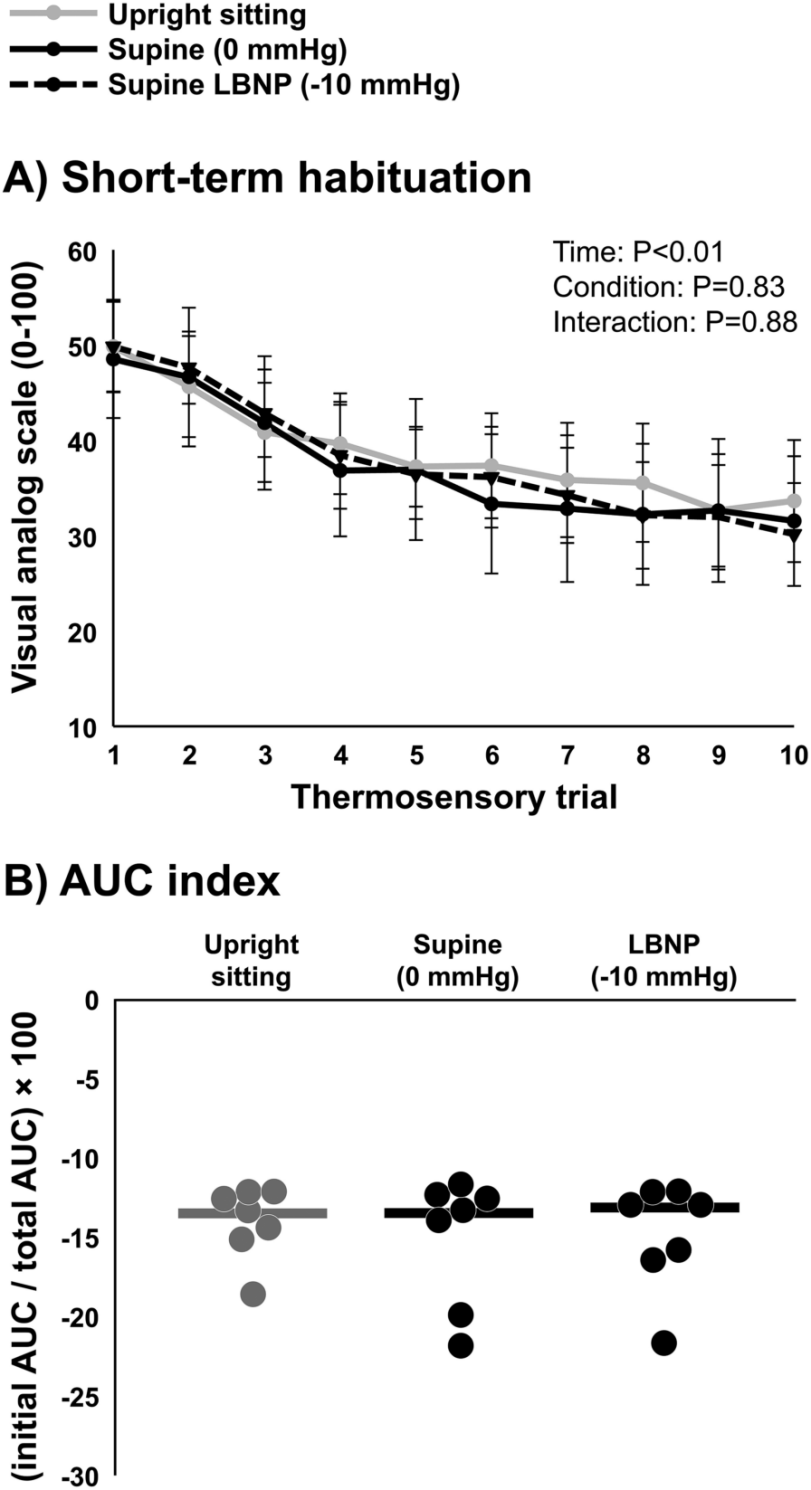
Short-term habituation during baroreceptor loading and unloading in young, healthy participants (*n* = 7). **(A)** Subjective pain ratings of ten repeated thermal stimuli in the upright sitting condition, supine position (control, 0 mmHg), and supine position with lower body negative pressure (LBNP) of −10 mmHg, and **(B)** short-term habituation during baroreceptor loading and unloading determined using the area under the curve method (AUC index). Potential differences between time points and conditions were tested using a two-way repeated measures ANOVA **(A)** and differences in AUC index between conditions were tested using a one-way repeated measures ANOVA **(B)**.

Average heat pain threshold was 42.8 ± 0.8°C. The average subjective pain rating for 105% of heat pain threshold was 50 ± 13 (VAS 0–100). A significant reduction in subjective pain ratings over the ten repetitive heat pulses was observed (*P* < 0.01), indicating short-term habituation (Figure 3A). Differences between time points and conditions were tested using two-way repeated measures ANOVA. Surprisingly, no major differences were detected in subjective pain ratings over the ten repetitive heat pulses when compared across conditions (upright sitting, LBNP 0 mmHg, and LBNP −10 mmHg) (*P* = 0.83). Similarly, no differences in short-term habituation were observed when calculating using the AUC index method (*P* = 0.58) (Figure 3B), and no differences were observed when calculating using the last-minus-first method (*P* = 0.57) or the slope method (*P* = 0.54). Differences in AUC index between conditions were tested using one-way repeated measures ANOVA. In summary, the young, healthy participants demonstrated significant short-term habituation, but this was unaffected by cardiopulmonary baroreceptor modulation.

### LBNP and short-term habituation in CBP patients (*n* = 12)

**Figure 4:**
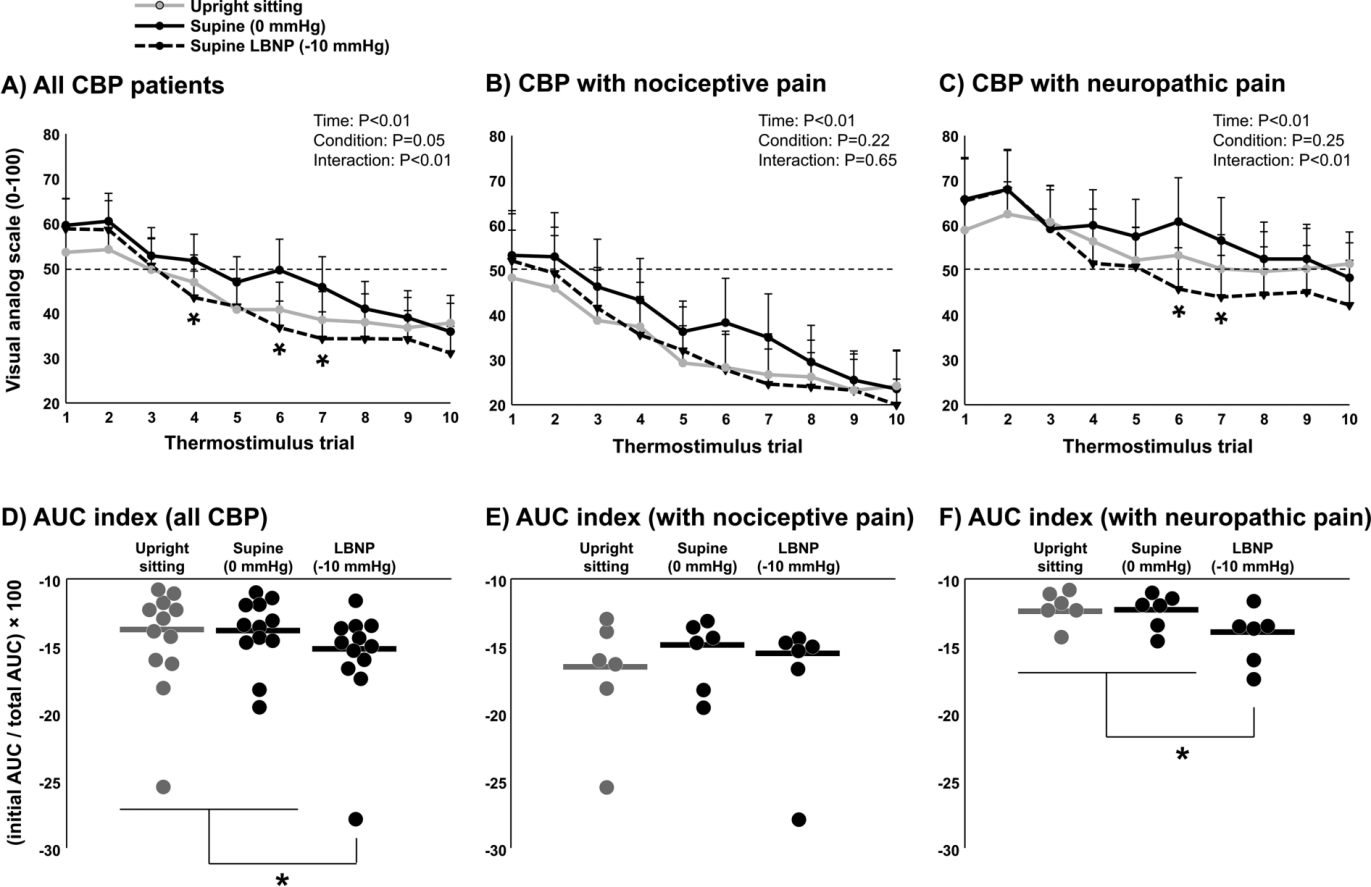
Short-term habituation during baroreceptor loading and unloading in patients with chronic back pain (CBP, *n* = 12). Subjective pain ratings of ten repeated thermal stimuli in the upright sitting condition, supine position (control, 0 mmHg), and supine position with lower body negative pressure (LBNP) of −10 mmHg in all CBP patients **(A)** and segregated into the CBP group with nociceptive pain components **(B)** (*n* = 6) and the CBP group with neuropathic pain components **(C)** (*n* = 6). Differences between time points and conditions were tested using a two-way repeated measures ANOVA. Short-term habituation during baroreceptor loading and unloading was determined using the area under the curve method (AUC index) in all CBP patients **(D)** and segregated into the CBP group with nociceptive pain components **(E)** and the CBP group with neuropathic pain components **(F)** Potential differences in AUC index between conditions were tested using a one-way repeated measures ANOVA. * *P* < 0.05.

Average heat pain threshold was 43.0 ± 1.3°C. The average subjective pain rating for 105% of threshold was 54 ± 20 (VAS 0–100). A significant reduction in subjective pain ratings over the ten repetitive heat pulses was observed (*P* < 0.01), indicating short-term habituation (Figure 4A). Differences between time points and conditions were tested using two-way repeated measures ANOVA. When comparing conditions (upright sitting, LBNP 0 mmHg, and LBNP −10 mmHg), a significant interaction was observed (*P* < 0.01) where subjective pain ratings were significantly lower during LBNP −10 mmHg compared with LBNP 0 mmHg. Interestingly, when considering only CBP patients with nociceptive pain components, no significant interaction was observed between conditions (*P* = 0.65) (Figure 4B). However, a significant interaction was observed between conditions (*P* < 0.01) when considering only CBP patients with neuropathic pain components (Figure 4C), suggesting that cardiopulmonary baroreceptor modulation of short-term habituation occurs in CBP patients with more severe symptoms. Indeed, short-term habituation among all CBP patients calculated using the AUC index method showed a significant increase during LBNP −10 mmHg compared with upright sitting and LBNP 0 mmHg (*P* = 0.02) (Figure 4D). However, CBP patients with nociceptive pain components did not demonstrate a significant increase in AUC index during LBNP −10 mmHg compared with upright sitting and LBNP 0 mmHg (*P* = 0.43) (Figure 4E), whereas CBP patients with neuropathic pain components did demonstrate a significant increase in AUC index during LBNP −10 mmHg compared with upright sitting and LBNP 0 (*P* = 0.04) (Figure 4F). Differences in AUC index between conditions were tested using one-way repeated measures ANOVA. Among the CBP patients with neuropathic pain components, short-term habituation was significantly different between conditions (upright sitting, LBNP 0 mmHg, and LBNP −10 mmHg) when calculated using the last-minus-first method (−9.8 ± 10.9 vs. −17.3 ± 9.4 vs. −23.2 ± 14.8 VAS 0–100, *P* = 0.05), but not when calculated using the slope method (−1.4 ± 1.4 vs. −1.9 ± 1.5 vs. −2.9 ± 2.0 VAS 0–100, *P* = 0.15). Among the CBP patients with nociceptive pain components, short-term habituation followed a similar pattern across conditions (upright sitting, LBNP 0 mmHg, and LBNP −10 mmHg) as the CBP patients with neuropathic pain components, but was not significantly different when calculated using the last-minus-first method (−23.4 ± 16.4 vs. −28.6 ± 18.9 vs. −32.1 ± 19.3 VAS 0–100, *P* = 0.21) or when calculated using the slope method (−2.9 ± 2.4 vs. −3.4 ± 2.4 vs. −3.6 ± 2.4 VAS 0–100, *P* = 0.38). Differences between conditions when using the last-minus-first method and slope method were tested using one-way repeated measures ANOVA.

### Pressure pain threshold

**Figure 5:**
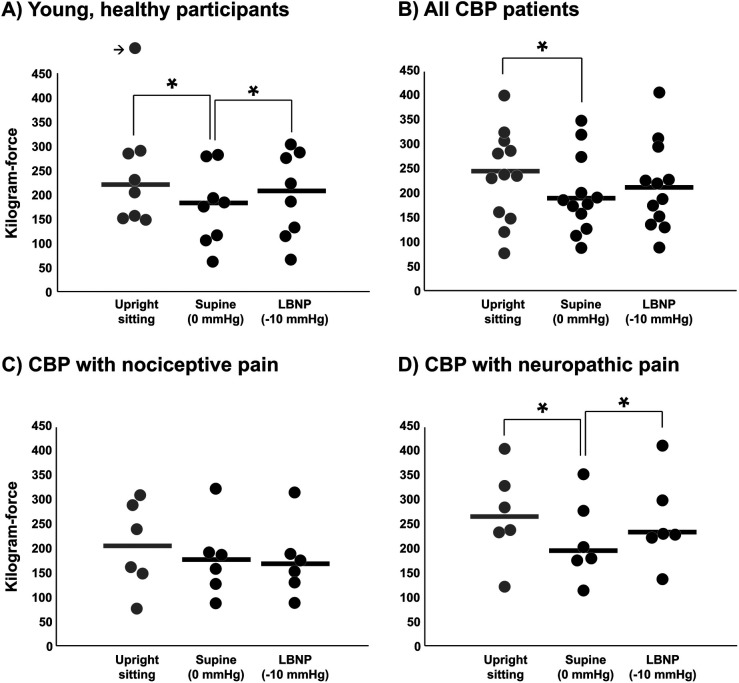
Mechanical pressure pain threshold during baroreceptor loading and unloading. Pressure pain threshold was tested in the upright sitting condition, supine position (control, 0 mmHg), and supine position with lower body negative pressure (LBNP) of −10 mmHg in young, healthy participants **(A)** (*n* = 8), in all CBP patients **(B)** (*n* = 12) and segregated into the CBP group with nociceptive pain components **(C)** (*n* = 6) and the CBP group with neuropathic pain components **(D)** (*n* = 6). The outlier data point in panel A is equal to 578 kgf and results were not different if removed. Potential differences between conditions were tested using a one-way repeated measures ANOVA. * *P* < 0.05.

Mechanical pressure pain threshold was also examined during LBNP to determine whether cardiopulmonary baroreceptor modulation of pain perception translates to other pain stimuli. In young, healthy participants, mechanical pressure pain threshold was significantly reduced in the supine position (LBNP 0 mmHg) compared with upright sitting (*P* < 0.01) (Figure 5A), demonstrating an increase in pain sensitivity by loading the cardiopulmonary baroreceptors with an increase in central blood volume. This effect of the supine position was reversed during LBNP −10 mmHg (*P* < 0.01). Similarly, CBP patients demonstrated a reduction in mechanical pressure pain threshold when moving from upright sitting to supine (LBNP 0 mmHg), but the change during LBNP −10 mmHg was not statistically significant for the entire group (Figure 5B). Like short-term habituation, an increase in pressure pain threshold was observed during LBNP −10 mmHg in CBP patients with neuropathic pain components (*P* = 0.01) (Figure 5D), whereas no significant change was observed among CBP patients with nociceptive pain components (*P* = 0.62) (Figure 5C). Differences between conditions were tested using one-way repeated measures ANOVA.

### Cardiovascular responses to LBNP

**Figure 6:**
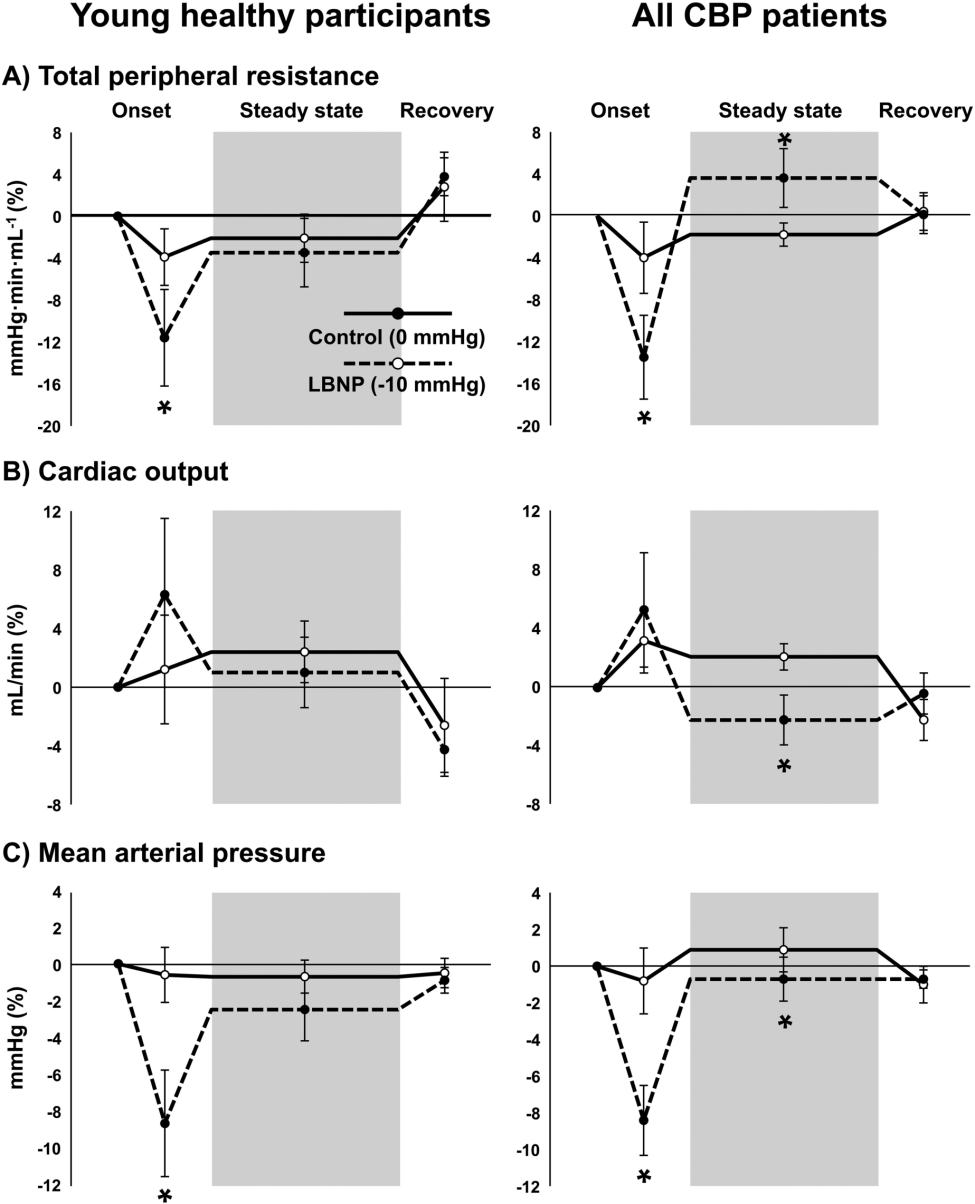
Cardiovascular responses to lower body negative pressure (LBNP). Total peripheral resistance **(A)**, cardiac output **(B)**, and mean arterial pressure **(C)** during the initial onset of LBNP, during steady state LBNP when sensory testing was performed (shaded region), and during recovery when chamber pressure reaches baseline (0 mmHg) in young, healthy participants (left panels) (*n* = 7) and patients with chronic back pain (CBP, right panels) (*n* = 12). Solid lines represent the control condition where no vacuum seal was present and chamber pressure remained at 0 mmHg, and dotted lines represent LBNP with chamber pressure of −10 mmHg (conditions were randomized). Because data were not always normally distributed, differences between timepoints and conditions were tested using Generalized Estimating Equations and pairwise comparisons were performed when significant interactions were detected. * *P* < 0.05 vs. control condition (0 mmHg).

As expected, total peripheral resistance (TPR) and mean arterial pressure (MAP) were significantly reduced during the 30-s onset of LBNP −10 mmHg compared with LBNP 0 mmHg in both CBP patients and young, healthy participants (Figure 6). However, during steady-state LBNP when sensory testing was performed, only CBP patients exhibited a significant increase in TPR and reduction in cardiac output (CO) and MAP compared with the control condition (LBNP 0 mmHg), suggesting reduced maintenance of CO and a greater reflex increase in sympathetic nerve activity. Indeed, the percent change in HR during steady-state LBNP −10 mmHg was higher among young healthy participants (7 ± 5 bpm) compared with CBP patients (2 ± 1 bpm) (*P* = 0.04). No significant differences in cardiovascular variables were observed during LBNP when comparing CBP patients with nociceptive and neuropathic pain components (all *P* > 0.05). Because data were not always normally distributed, differences between timepoints and conditions were tested using Generalized Estimating Equations (GEE).

## Discussion

The present study examined short-term pain habituation to a repetitive thermal stimulus in patients with CBP using a protocol that first demonstrated good reliability in young, healthy adults. There are several novel findings. First, like previous work (53), short-term habituation was observed in CBP patients and in young, healthy participants. Second, despite significantly reduced conditioned pain modulation in CBP patients with neuropathic pain components, we observed the largest increases in short-term habituation during LBNP among CBP patients with neuropathic pain components. Third, LBNP modulation of pain perception was also observed during mechanical pressure pain in CBP patients, suggesting that cardiopulmonary baroreceptors can modulate perception of various pain modalities. These data highlight a significant impact of chronic pain on baroreceptor control of nociception and provide a novel protocol to examine short-term pain habituation in this patient population.

### Descending pain inhibition

Conditioned pain modulation, which tests the efficacy of a noxious conditioning stimulus to reduce pain perception of a simultaneous noxious stimulus, has been the primary descending pain inhibition pathway studied clinically and experimentally (38). Meta-analyses provide strong evidence that conditioned pain modulation is impaired in populations with chronic pain (39, 54). More specifically, studies including patients with features of neuropathic pain more frequently report a correlation between conditioned pain modulation and pain intensity than studies focused on nociceptive pain (54). Indeed, we demonstrated reduced conditioned pain modulation in patients with features of neuropathic pain compared with nociceptive pain using a standard conditioned pain modulation paradigm (39) (Table 1). For this reason, we rationalized that greater severity of chronic pain (i.e., with neuropathic pain components) would limit cardiopulmonary baroreceptor modulation of short-term habituation because of reduced potential for descending inhibition of pain. However, we observed the opposite where CBP patients with nociceptive rather than neuropathic pain features were limited in the increase in short-term habituation during cardiopulmonary baroreceptor unloading. These results are corroborated by the findings of limited increases in mechanical pressure pain threshold during cardiopulmonary baroreceptor unloading in CBP patients with nociceptive pain components compared with nociceptive components. Together, these findings pose important clinical implications if they can be corroborated by larger studies because they suggest cardiopulmonary baroreceptors could potentially be targeted for pain management in patients with more severe symptoms of chronic pain.

### Potential mechanisms of LBNP-induced analgesia

LBNP performed at −10 mmHg, as was performed in the present study, elicits a significant reflex increase in sympathetic nerve activity of approximately +25% (45–47) proportional to the reduction in stroke volume (47, 55, 56). That said, there is evidence for reduced pain perception during elevations in sympathetic nerve activity in healthy humans (57) without involvement of the endogenous opioid system (58). Dayan et al. (57) reported a correlation between the increase in sympathetic tone (increase in total peripheral resistance) and pain adaptation following administration of yohimbine, which is an *α*_2_-adrenergic receptor antagonist. More recently, Makovac et al. performed functional MRI studies and reported that the association between autonomic activity and descending inhibition is mediated primarily by the functional connectivity between the periaqueductal grey and the ventro-medial prefrontal cortex (59). Results from the present study extend these findings by demonstrating analgesic effects of sympathetic activation via cardiopulmonary baroreceptor unloading.

Previous studies examining the cardiopulmonary baroreceptor control of pain provide evidence that elevated BP influences the analgesic response (17–19). Although CBP patients in the present study were generally not hypertensive based on ambulatory 24-hour BP monitoring, supine BP tended to be higher in the CBP patients with features of neuropathic pain compared with CBP patients with nociceptive pain (Table 1), in addition to a greater increase in short-term habituation during LBNP. Although no correlation was observed between BP and short-term habituation (data not shown), these findings are in line with previous studies (17, 18). For example, in men with normal BP, higher ratings of heat pain were reported during passive leg elevation (baroreceptor loading) (18). However, in men with elevated BP, ratings of a heat pain stimulus were reduced during passive leg elevation (baroreceptor loading) (18) and when using a painful finger pressure stimulus (17). This discrepancy in results when comparing groups of men with normal vs. elevated BP may be explained by findings from Mark et al. who showed that cardiopulmonary baroreceptor sensitivity is enhanced using mild lower body negative pressure (e.g., −10 mmHg) in individuals with borderline hypertension (60). Therefore, considering the increased supine BP among CBP patients with neuropathic features in the present study, it is tempting to speculate that these participants had higher cardiopulmonary baroreceptor sensitivity and therefore a more robust analgesic response to cardiopulmonary baroreceptor unloading. However, this speculation would warrant further studies that include CBP patients with and without established hypertension.

Conditioned pain modulation using cold water was present among patients with features of nociceptive pain whereas cardiopulmonary baroreceptor-mediated modulation was present among those with neuropathic pain. Although both modalities involve descending inhibition, the lack of correlation between the two modalities is not surprising given that conditioned pain modulation involves higher-level cortical descending systems and is affected by psychological factors (emotion, cognition, and attention), whereas cardiopulmonary baroreceptor-mediated modulation involves lower-level brainstem circuits not under direct influence of psychological factors. The reduction in conditioned pain modulation among patients with features of neuropathic pain may potentially be explained, in part, by diminished endogenous opioid signaling. The endogenous opioid system has shown to be involved in the conditioned pain modulation paradigm used in the present study (61) and impaired in neuropathic pain conditions (62).

### Methodology for eliciting short-term habituation

Adaptation to pain stimuli is dependent on an aggregate of parameters, such as stimulus frequency, intensity, rate of increase/decrease, and anatomical location. Regarding the stimulus frequency, habituation to heat pain has been reported using relatively long duration intervals between stimuli, such as up to 80 s (36) or 20 s (53). When using a short duration interval, such as ≤3 s, sensitization rather than habituation can be observed (34, 35), and the increase in perceived pain during repeated stimuli with intervals of ≤3 s is associated with C nociceptor fiber discharge (36, 63). However, in the present study, short intervals of 2 s elicited short-term habituation. The reason for the discrepancy may be related to the stimulus intensity. Instead of using a relatively high stimulus intensity that can elicit wind-up (e.g., 53°C) (35), a lower stimulus intensity equal to 105% of heat pain threshold was utilized (∼44°C–46°C). These findings are in line with a previous study (53) demonstrating habituation to a heat stimulus when repeatedly testing heat pain threshold in patients with chronic pain and healthy individuals. However, although the interval between stimuli was not reported, it was significantly longer than the present study because each stimulus was a ramp from 32°C to heat pain threshold (53).

The rate of temperature increase also plays a significant role in eliciting habituation. Studies have failed to elicit habituation in chronic pain when using relatively slow rates of temperature increase during stimulation (0.5°C/sec) (4), whereas studies using faster rates of temperature increase successfully elicited habituation (1.5°C/sec) (53). Notably, peak intensity was equivalent to heat pain threshold (4, 53). The present study included a faster rate of temperature increase, which averaged ∼2.0°C–2.5°C/sec, depending on the participant's heat pain threshold (see methods). Thus, greater rate of temperature increase may open the opportunity for habituation if the peak heat intensity is near pain threshold.

### Methodology for quantifying short-term habituation

Several approaches were used to quantify short-term habituation during the repetitive stimuli, such as the slope of the line fitted to the series of 10 stimuli (slope method) (34), the difference between the ratings of the first and last stimuli (last-minus-first method) (49), and the area under the curve (AUC index). All three approaches demonstrated good reliability (Figure 1E). However, the slope method and last-minus-first method assume a linear change in pain perception during a repetitive stimulus and may not be sensitive to the steep, non-linear change observed within the first several stimuli. The AUC index approach has the distinct advantage of quantifying non-linear short-term habituation and accounts for the baseline heat pain sensitivity. For example, if two individuals have an equal starting point (initial AUC), the individual with a more rapid, non-linear decline in pain perception will demonstrate lesser total AUC and therefore greater short-term habituation. Therefore, although these different approaches are reliable and sufficient (slope, last-minus-first method, and AUC index), the AUC index approach is recommended for quantifying short-term habituation because it appears more sensitive to the non-linear reduction in pain perception over the repeated stimuli.

### Limitations

A primary limitation of the study was applying the heat stimulus only to the anterior forearm. Adaptation to the heat stimulus is likely body-site-specific and may not occur at a different anatomical site. The second limitation was not including a group of healthy adults without chronic pain that were age-matched to the CBP patients for direct comparisons. This is important because sensitization to thermal pain increases with age (64). The group of young, healthy participants included in the study was for the purpose of determining reliability of the protocol, not for direct comparison with the significantly older group of CBP patients. The third limitation is reliance on self-report questionnaires for characterization of nociceptive vs. neuropathic pain qualities. Because quality of pain is subjective, measurements must rely on the patients’ self-report. Third, cardiopulmonary baroreceptor sensitivity was not directly tested in the present study. Although it is known that cardiopulmonary baroreceptor sensitivity changes with hypertension status, it remains unclear whether cardiopulmonary baroreceptor sensitivity changes in conditions of chronic pain.

### Perspectives

Traditionally, it has been believed that baroreceptors reduce pain perception only when activated—such as during periods of elevated arterial pressure (65). However, the findings from the present study challenge this long-standing view. Here, we observed decreased pain perception—reflected by enhanced short-term habituation—when baroreceptors were *deactivated* (unloaded), rather than activated. The discrepancy may be a result of chronic pain-related alterations in autonomic function. While the clinical relevance of this finding is not immediately clear—especially since LBNP, the method used to unload baroreceptors, is rarely employed in medical practice—it opens the door to intriguing possibilities. One such possibility lies in the hemodynamic effects of anti-hypertensive therapies, which may mimic certain aspects of LBNP. For example, diuretics reduce plasma volume and consequently decrease the stretch of cardiopulmonary baroreceptors. Yet, despite their widespread use, there is a surprising lack of research examining whether diuretics influence pain perception in patients with chronic pain. This gap highlights an interesting avenue for future investigation into the potential analgesic effects of commonly used cardiovascular medications.

## Conclusion

In summary, the present study demonstrates short-term pain habituation to a repetitive thermal stimulus in patients with CBP using a novel protocol with reliability, and further demonstrate a significant increase in short-term habituation in CBP via cardiopulmonary baroreceptor unloading. Interestingly, significant increases in short-term habituation during LBNP were observed primarily among CBP patients with neuropathic pain components. Although these results do not conclusively demonstrate a causal link between cardiopulmonary baroreceptors and analgesia, our findings do extend the growing body of literature featuring baroreflex-mediated analgesia to include cardiopulmonary baroreceptors in patients with CBP and a role for short-term habituation.

## Data Availability

The raw data supporting the conclusions of this article will be made available by the authors upon reasonable request.
